# Recent Patents of Interest to Dentists

**Published:** 1907-05

**Authors:** 


### PATENTS.

### RECENT PATENTS OF INTEREST TO DENTISTS.

844781. Dental Gold Pellet Holder, Henry P. Davis, Washington, D. C.
844395. Tooth Brush, Edward Penkala, Zagrab, Austria-Hungary.
845064. Tooth Brush, Frederick P. Drowne, Warren, R. I.
846030. Instrument Tray, Wm. G. Hullhorst, Toledo, Ohio.
846420. Dental Bracket, Albert W. McKenney, Roxbury, Mass.
845822. Head-Rest for Chairs, David Sarason, Berlin, Germany.
845835. Gas Regulator for Dental Vulcanizers, Elmer E. Wightman, Chicago, Ill.
846900. Tooth Brush, Wm. O. Bloom, Worcester, Mass.
846998. Pyrometer, John F. Hammond, Brewster, N. Y.
847778. Tooth Separator, James W. Ivory, Philadelphia, Pa.
847591. Dental Handpiece. Henry S. Miller, Rochester, N. Y.
847828. Toothpick Holder. Wilhelm Sandleben, Hamburg, Germany.
848001. Head Rest for Barber and Other Chairs, Eugene Berninghaus, Cincinnati, Ohio.
848403. Dental Obtunder. Perry R. Skinner, Amsterdam, N. Y.
848406. Tooth Pick Machine. Willis W. Tainter and C. P. Stanley, Dixfield, Mo.
848334. Combination Dental Plugger. Percy E. Williams, Savannah, Ga.

849208. Dental Handpiece. Lyter H. Crawford, New York, N. Y.
849209. Combination Mouth Mirror and Lamp, Lyter H. Crawford, New York, N. Y.
849335. Electric Dental Furnace. Lewis Markwitz, San Francisco, Cal.
848693. Toothpick Holder. Charles H. Sharp, Lockport, N. Y.
848863. Reinforce and Backing for Artifical Teeth. Isidore Stern, New York, N. Y.
849297. Dental Swaging Device. George J. Weber, Liberty Center, Ohio.
849595. Head Rest for Barbers Chairs. Benjamin F. Buchanan, Camden, N. J.
849702. Guard and Moistener for Dental Tools. Charles A. Sevier. Jackson, Tenn.
850661. Dental Engine. Jesse A, Lewis, Elgin, Nebr.

Copies of above patents may be obtained for fifteen cents each by addressing John A. Saul, Solicitor of Patents, Fendall Building, Washington, D. C.

